# Visual input regulates melanophore differentiation

**DOI:** 10.3389/fcell.2024.1437613

**Published:** 2024-08-20

**Authors:** Karen Atkinson-Leadbeater, Gabriel E. Bertolesi, Sarah McFarlane

**Affiliations:** ^1^ Department of Psychology, Mount Royal University, Calgary, AB, Canada; ^2^ Department of Cell Biology and Anatomy, Hotchkiss Brain Institute, Alberta Children’s Hospital Research Institute, University of Calgary, Calgary, AB, Canada

**Keywords:** differentiation, *Xenopus laevis*, melanophore, melanin, melatonin, TYRP1, pigmentation

## Abstract

**Introduction:**

Developmental processes continue in organisms in which sensory systems have reached functional maturity, however, little research has focused on the influence of sensory input on cell and tissue development. Here, we explored the influence of visual system activity on the development of skin melanophores in *Xenopus laevis*.

**Methods:**

Melanophore number was measured in X. laevis larvae after the manipulation of visual input through eye removal (enucleation) and/or incubation on a white or black substrate at the time when the visual system becomes functional (stage 40). To determine the developmental process impacted by visual input, migration, proliferation and differentiation of melanophores was assessed. Finally, the role of melatonin in driving melanophore differentiation was explored.

**Results:**

Enucleating, or maintaining stage 40 larvae on a black background, results in a pronounced increase in melanophore number in the perioptic region within 24 h. Time lapse analysis revealed that in enucleated larvae new melanophores appear through gradual increase in pigmentation, suggesting unpigmented cells in the perioptic region differentiate into mature melanophores upon reduced visual input. In support, we observed increased expression of melanization genes *tyr*, *tyrp1*, and *pmel* in the perioptic region of enucleated or black background-reared larvae. Conversely, maintaining larvae in full light suppresses melanophore differentiation. Interestingly, an extra-pineal melatonin signal was found to be sufficient and necessary to promote the transition to differentiated melanophores.

**Discussion:**

In this study, we found that at the time when the visual system becomes functional, *X. laevis* larvae possess a population of undifferentiated melanophores that can respond rapidly to changes in the external light environment by undergoing differentiation. Thus, we propose a novel mechanism of environmental influence where external sensory signals influence cell differentiation in a manner that would favor survival.

## 1 Introduction

Development requires the coordinated regulation of cell proliferation, specification, migration, differentiation and morphogenesis. Because embryonic development largely occurs prior to the emergence of sensory systems, research has concentrated on mechanisms internal to the organism that drive these events, revealing inducers and transcription factors that guide tissue development. However, development continues in organisms with mature sensory systems and the corresponding abilities to detect external stimuli, raising the possibility that sensory input influences developmental events.

Sensory experience plays an important role in the developing nervous system. A striking example is the effect of ocular deprivation on developing mammalian visual circuits (reviewed in Katz and Shatz, 1996), where blocking sensory input from one eye dramatically reduces the responsiveness of cortical neurons driven by the deprived eye (Wiesel and Hubel, 1963). Altered cellular morphology is a key mechanism by which sensory experience influences neural circuits (Knudsen, 2004). For instance, sensory experience promotes axonal arborization and synaptogenesis of geniculocortical neurons (Antonini and Stryker, 1993), and the appropriate song stimuli significantly decreases dendritic spine density of zebra finch neostriatal neurons (Wallhausserfranke et al., 1995). Sensory input also impacts zebrafish neurogenesis; visual input stimulates survival of late born optic tectum neurons (Hall and Tropepe, 2018b), and swimming-induced sensory input promotes forebrain neural precursor self-renewal (Hall and Tropepe, 2018a). Thus, sensory experience influences nervous system development. However, beyond the nervous system, we know little about environmental control of development.

The nervous system allows organisms to respond to environmental stimuli, as such it is appropriate that its development is influenced by the external environment. Intriguingly, melanophores, melanin-producing cells in poikilotherms, also respond to environmental stimuli. Changes in pigmentation contribute to organismal survival through a number of mechanisms that include camouflage, temperature regulation and UV protection (Nilsson Sköld et al., 2013). For instance, changes in environmental light/dark conditions rapidly disperse and aggregate melanin-filled vesicles (melanosomes) inside melanophores to increase or decrease larval skin pigmentation, respectively. In contrast, longer-term light challenges alter pigment cell numbers through a mechanism that relies on the visual system and not on the light-sensing pineal complex or melanophores (Uchida et al., 2000; Sugimoto, 2002; Bertolesi et al., 2016a; Bertolesi et al., 2016b). Thus, the external light environment can influence melanophore number, however, the mechanism by which this occurs is unknown.

Melanophores arise from the neural crest through cascading stages of differentiation that appear to occur independently of environmental influence. Several secreted signals, including bone morphogenetic proteins, fibroblast growth factors, and Wnt/β-catenin, interact to establish the neural plate border, marked by the expression of transcription factors such as *pax3* and *zic1*, which then work in concert to drive *snai2* and *sox10* expression in neural crest cells (Wu et al., 2003; Hong and Saint-Jeannet, 2007; Pegoraro and Monsoro-Burq, 2013; Pla and Monsory-Burq, 2018). Subsequently, Pax3, Sox10, and Wnt/β-catenin signaling cooperate to drive the expression of Micropthalmia-associated transcription factor (Mitf) (Mort et al., 2015; Sutton et al., 2021). Mitf in turn promotes the expression of effector genes involved in pigmentation including melanin synthesis enzymes *tyrosinase* (*tyr*), *tyrosinase-like protein 1* (*tyrp1*), and *tyrp*2 (formerly known as *dopachrome tautomerase*), and the melanosome biogenesis protein *pre-melanosome protein* (*pmel*), a structural matrix protein upon which melanin granuels are deposited (Baxter and Pavan, 2003; Kumasaka et al., 2005; Kawakami and Fisher, 2017). In *X. laevis*, the first *mitf*-positive cells appear in the dorsal midline of the late neurula (Kumasaka et al., 2004). *tyr*, *tyrp1*, and *tyrp2* positive cells emerge subsequently (Kumasaka et al., 2003), such that mid-tailbud stage embryos are pigmented (Frunchak and Milos, 1990). With the emergence of a functional visual system at the end of embryogenesis (Tao et al., 2001; Bertolesi et al., 2014), light can then influence melanophore numbers: melanophore numbers increase in enucleated or dark-reared *X. laevis* larvae (Bertolesi, et al., 2016a; Bertolesi, et al., 2016b). Importantly, the developmental process(es) being controlled by light signaling has not been identified.

Here, we examine the mechanism by which the light environment influences melanophore development in *X. laevis* larvae, at the stage when many pigmented melanophores are present and the visual system is active. We find that within a day, enucleated larvae or larvae moved to a black background show a dramatic increase in pigmented cells unique to the perioptic region. Interestingly, this increase arises through the differentiation of melanophore precursors and not through proliferation or pigment cell migration. Specifically, un-pigmented perioptic melanophore precursors turn on melanization genes including *tyrp1*, *tyr*, and *pmel*. Intriguingly, this undifferentiated, unpigmented population appears to be held at the ready in an immature state when larvae are maintained in the light on a white background. Neural information from the visual system, rather than an eye-derived paracrine signal, is necessary for enucleation-induced differentiation. Finally, a melatonin signal is necessary for and sufficient to induce this rapid melanization. Overall, we propose that visual input controls the differentiation of melanophores, thus describing a novel mechanism by which environmental stimuli impact cell maturation.

## 2 Materials and methods

### 2.1 *Xenopus laevis* embryos

The University of Calgary and Mount Royal University Animal Care and Use Committees approved all procedures involving frogs, embryos and larvae, which were maintained at the University of Calgary. Adult *X. laevis* females were injected with human chorionic gonadotropin (Intervet Canada Ltd.) to stimulate egg production. Eggs were fertilized *in vitro* and maintained in a 0.1X Marc’s Modified Ringer (MMR; 0.1 M NaCl, 2 mM KCl, 1 mM MgCl_2_, 2 mM CaCl_2,_ 5 mM HEPES, pH7.5) solution at 16°C for 5 days, until they reached stage 40 (staging of embryos according to Nieuwkoop and Faber (1994); Sive and Harland (2000)). Embryos were exposed to light and dark environments from fertilization to stage 40 except where otherwise indicated. Embryos were randomly selected for experiments, and rare embryos with abnormal morphology or pigmentation levels were excluded.

### 2.2 Enucleation, optic nerve transection, and lighting conditions

Stage 40 *X. laevis* embryos were collected and anesthetized using modified Barth’s Solution (MBS; 8.8 mM NaCl, 0.1 mM KCl, 0.7 mM MgSO_4_, 5 mM HEPES, pH 7.8, and 25 mM NaHCO_3_) supplemented with 0.4 mg/mL tricaine (Sigma-Aldrich). Embryos were subjected to bilateral enucleation, or sham surgery. The enucleation procedure used a pin tool to make a small incision through the skin and dura along the posterior ventral rim of the eye. The eye was pushed out through the incision and the optic nerve severed using forceps. For the sham surgery, the incision was generated, but the eye was left in place.

Following surgery, larvae were incubated for 24 h at 21°C–23°C in 0.1X MMR under specific lighting: sham surgery larvae were maintained in the light with a white background (control) or light with a black background, and enucleated larvae were maintained in the light on a white background. To produce white and black backgrounds, dishes were placed over white or black corrugated plastic sheets, respectively. Overhead lighting was provided by a single 12-inch fluorescent tube suspended 10 inches above the dishes. Embryos underwent a 30 min exposure to melatonin (100 μM; Sigma-Aldrich) prior to fixation to aggregate pigment granules and facilitate cell couting (see below). Embryos and larvae were fixed in either 4% paraformaldehyde (PFA) for melanophore counting, or in MEMFA (1X MEM salts [0.1 M MOPS, 2 mM EGTA, 1 mM MgSO_4_], 1% formaldehyde in RNAse-free water) for whole mount *in situ* hybridization (WMISH). Occasionally, 0.003% (w/v) 1-phenyl-2-thiourea (PTU), which reduces tyrosinase activity (Karlsson et al., 2001), was included for the 24 h exposure period to limit new pigment production and possible interference with ISH staining in the perioptic area.

For optic nerve transection, the eyes first underwent GFP electroporation to label the optic nerve. Briefly, stage 28 embryos were anesthetized (MBS + tricaine) and placed dorsal side up in a Sylgard-filled Petri dish. Approximately 5 nL of a 250 ng/μL pCS2-GFP plasmid DNA solution was injected into the space between the eye and brain and ten 50 ms 50 mV pulses, spaced one second apart were delivered by a Grass S44 stimulator through platinum electrodes placed on either side of the head (Haas et al., 2002; Atkinson-Leadbeater et al., 2016). Embryos with a clearly GFP-labeled optic tract were selected at stage 40 and placed in one of the following conditions: sham surgery controls; double enucleation; single enucleation, where the non-electroporated eye was removed; and single enucleation with optic nerve transection, where the non-electroporated eye was removed and the GFP-positive optic nerve from the electroporated eye was severed. The optic nerve was transected with a pin tool inserted through a small incision through the skin and dura along the edge of the eye. All larvae were maintained at room temperature in the light on a white background for 24 h at which time larvae were treated with melatonin and fixed in 4% PFA for melanophore counting. GFP negative optic nerve and optic tectum indicated a successful transection, and pigment cells were counted in a blinded fashion (see below).

### 2.3 Pigment cell number

**FIGURE 1:**
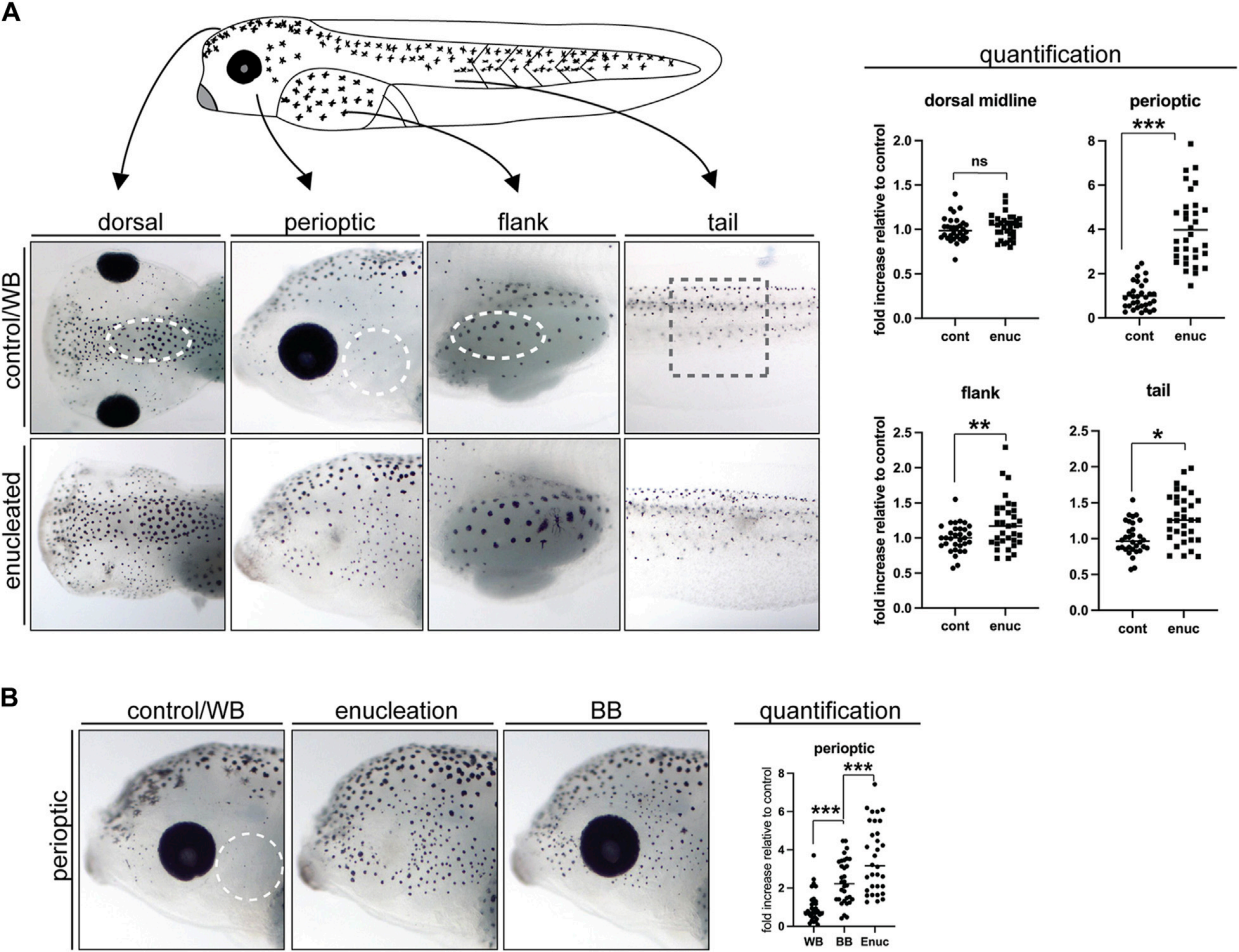
Enucleation and exposure to a black background dramatically increase perioptic melanophore numbers. **(A)** The position and size of the counting domains (dashed lines) within each region of analysis is indicated: dorsal midline, perioptic area, flank, and tail (see methods for details). In comparison to controls, melanophore number was unchanged along the dorsal midline, increased dramatically in the perioptic zone, and minimally increased in the flank and tail of enucleated larvae. (dorsal midline: $\bar{X}$ _*cnt*_ = 1.00 ± 0.02, $\bar{X}$ _*enuc*_ = 1.03 ± 0.02; perioptic: $\bar{X}$ _*cnt*_ = 1.00 ± 0.11; $\bar{X}$ _*enuc*_ = 3.98 ±0.28; flank: $\bar{X}$ _*cnt*_ = 1.00 ± 0.04; $\bar{X}$ _*enuc*_ = 1.20 ± 0.06; tail: $\bar{X}$ _*cnt*_ = 1.00 ± 0.04, $\bar{X}$ _*enuc*_ = 1.29 ± 0.06; mean ± s.e.m.; *n*_*cnt*_ = 32, *n*_*enuc*_ = 33; *N* = 4) **(B)** The impact of 24 h exposure to a black background (BB) on perioptic melanophore number was examined. When compared to white background (WB)-exposed controls, BB-treated larvae showed a significant increase in perioptic melanophores, although enucleated larvae displayed an even greater increase in these cells. ( $\bar{X}$ _*cnt/WB*_ = 1.00 ± 0.13, $\bar{X}$ _*BB*_ = 2.34 ± 0.20, $\bar{X}$ _*enuc*_ = 3.56 ± 0.32; F_*3,96*_ = 30.42, *p < 0.0001*, one-way ANOVA) n. s., not significant, *p < 0.05, **p < 0.001, ***p < 0.0001, unpaired two-tailed Student’s *t*-test **(A)**, and Tukey’s **(B)**.

For pigment cell counting, cells were treated briefly with melatonin prior to being fixed to aggregate the melanosomes, which allowed for easy identification of individual melanophores as each melanophore contained a single “dot” of pigment. Fixed embryos were imaged at 20 to 40 times magnification using a Lumenera Infinity HD microscope camera mounted on an Olympus SZ61 stereomicroscope and the Infinity HD software. The appropriate region was selected from each image in Adobe Photoshop, copied, and randomly arranged on a grid so that counting could be performed blind to the treatment conditions. Counting was performed in specific zones (Figure 1): for the dorsal head an oval domain positioned along the dorsal midline at the posterior edge of the head; for the perioptic zone, a circular region positioned between the eye and the flank (yolk sac); for the abdominal region an oval domain positioned along the lateral midline at the anterior end of the developing abdomen; for the tail a square positioned just posterior to the cloaca. The dimensions of the selection zones were kept consistent between groups within an experiment for comparison purposes. To account for pigmentation differences between clutches, for each experiment melanophore numbers in enucleated larvae were normalized to those in sibling controls subjected to a sham surgery and maintained in the light on a white background. Even with normalization, differences were observed in the fold increase in pigment cell numbers between clutches, however, the direction of the effect was always maintained.

### 2.4 RNA *in situ* hybridization

Probe synthesis and WMISH were largely performed as described previously (Sive and Harland, 2000). In order to capture both the pigment and the WMISH label, yet to maintain larvae in RNAse-free conditions, larvae were imaged during the wash period after the incubation in the alkaline-phosphatase (AP) conjugated anti-digoxygenin (DIG) antibody (Roche). The colorimetric reaction introduced 5-bromo-4-chloro-3-indoyl phosphate (BCIP; Roche) and nitro blue tetrazolium (NBT; Roche) in an alkaline phosphatase buffer (100 mM Tris (pH 9.5), 50 mM MgCl_2_, 100 mM NaCl, 0.1% Tween 20, 2 mM levamisole (Sigma-Aldrich) (Sive and Harland, 2000). The insoluble substrate produced by the AP-catalyzed reaction was somewhat masked by the melanosome aggregates in melanophores, therefore, larvae were incubated overnight in a bleaching solution (1% H_2_O_2_, 5% formamide, 0.5x SSC; 20x SSC: 3 M NaCl, 0.3 M sodium citrate) on a fluorescent light box to reveal the extent of staining. At this point, larvae were re-imaged, and the images were matched against the initial pigment images using the unique patterns of melanophores present in each larva.

The *tyr* and *tyrp1* probes were synthesized as described previously (Kumasaka et al., 2003; Bertolesi, et al., 2016b). All probes were generated by RT-PCR of cDNA from whole embryos between stages 39 and 42 using SuperScriptTM II RNase H reverse transcriptase (Invitrogen). PCR products were cloned into pCRII TOPO and sequenced at the Sanger Sequencing facility at the University of Calgary, Canada. DIG-labeled single stranded RNA probes were synthesized using linearized plasmid. The primers used were as follows: *tyr* forward (5′-aagatggcctctggagaaacg) reverse (5’-ccatgggctccgtttcataat); *tyrp1* forward (5′-tcaccccacaggctctacaaa) reverse (5′-tgcagatgtcacattggttgc); *mitf* forward (5′-gcagatgcaacagcaagagcg) reverse (5′-aacccagcttggtggicgatac); and *pmel* forward (5′-gtcgtcctttccctcgtggtg) reverse (5′-gcttggttgggttcagcctcttc). The amplicon sequences obtained corresponded to the following GenBank Accession numbers: XM 041582724.1 (from nucleotide 894 to 1317), NM 001087023.1 (from nucleotide 165 to 860), NM 001172175.1 (from nucleotide 131 to 1090), and XM 018247403.2 (from nucleotide 629 to 1739), for *tyr*, *tyrp1*, *mitf*, and *pmel* probes, respectively.

### 2.5 Cell differentiation state analysis

For the experiments depicted in Figure 3, larvae were imaged after WMISH and the number of expressing cells were counted in the perioptic region (dashed white line). For Figure 4, larvae were imaged prior to and after the colorimetric reaction in the WMISH protocol to capture pigmentation and *tyrp1* mRNA expression, respectively. Perioptic and flank domains were cropped out of pigment and ISH images (as described above). The patterns of melanophores were sufficient to capture the same domains from each image, and these were overlayed in Adobe Photoshop to allow the identification and quantification of each cell type (pigmented and *tyrp1*+, *tyrp1*+ only, pigmented only). The schematics in Figure 4 were generated by tracing and overlaying the outlines of the pigmentation and *tyrp1* mRNA probe in Adobe Illustrator.

### 2.6 Inhibition of cell proliferation

Stage 40 *X. laevis* larvae (control or enucleated) were incubated in a control 4% dimethyl sulfoxide (DMSO; Sigma-Aldrich) or a 150 μM aphidicolin (Sigma-Aldrich) and 20 mM hydroxyurea (Sigma-Aldrich) solution in 0.1X MMR for 24 h at room temperature prior to melatonin treatment and fixing in 4% PFA. Importantly, this cocktail has been used previously to inhibit DNA replication and block cell proliferation in *Xenopus* and zebrafish embryos (Harris and Hartenstein, 1991; Cechmanek and McFarlane, 2017). To assess proliferation, larvae were subjected to whole mount immunohistochemistry using a rabbit polyclonal anti-phosphohistone H3 antibody (Millipore, Cat# 06–570, Lot#3193845), which labels mitotic cells in *X. laevis* (McKeown et al., 2013). Larvae were imaged under fluorescence and brightfield conditions using the QIClick monochrome CCD camera mounted on an Olympus BX41 fluorescent microscope and CellSens software (Olympus). The relative change in pHH3 positive cells and pigment positive cells across groups was assessed in a blinded fashion as described above.

### 2.7 Time-lapse imaging of pigment cell emergence

Stage 40 larvae that underwent enucleation surgery were maintained at room temperature in individual 35 mm Petri dishes containing 0.1X MMR in the light on a white background. Nine hours after surgery, larvae were exposed to tricaine in MBS for 5 minutes to anesthetize the larvae and to induce pigment granule aggregation. After imaging, larvae were rinsed and returned to 0.1X MMR. This procedure was repeated every three to 4 hours over the next 10 hours. For analysis, a common area for each time point image was identified and all melanophores within the area were tracked and accounted for so that new melanophores could be identified.

### 2.8 Area measurement of pigment granule aggregates

Melatonin treatment results in the aggregation of melanosomes into a single ‘dot’ (or aggregate) within each melanophore, with the size of each aggregate reflecting the quantity of melanin present within each pigment cell. ImageJ software (NIH) was used to measure the pixel area of the aggregated melanosomes for each cell within the counting zone in the perioptic and flank regions. An average melanosome aggregate area was calculated for each embryo and subsequently used to calculate the average aggregate area for each condition. To account for inter-clutch differences in baseline melanin production, areas were normalized to the controls (larvae maintained in the light on a white background) within each experiment.

### 2.9 24 h melatonin and 4-phenyl-2-propionamidotetralin (4P-PDOT) exposure

Stage 40 *X. laevis* larvae (control or enucleated) were incubated in a 0.5% ethanol, 100 μM melatonin (Sigma-Aldrich), or 50 μM 4P-PDOT (Tocris Bioscience, Burlington, ON, Canada) solution in 0.1X MMR for 24 h at room temperature prior to fixing in 4% PFA. Larvae were maintained under overhead lighting on a white background (WB and enucleation), or black background (BB) during exposure period, and imaged following fixation for perioptic melanophore number assessment.

### 2.10 Statistical methods

**FIGURE 2:**
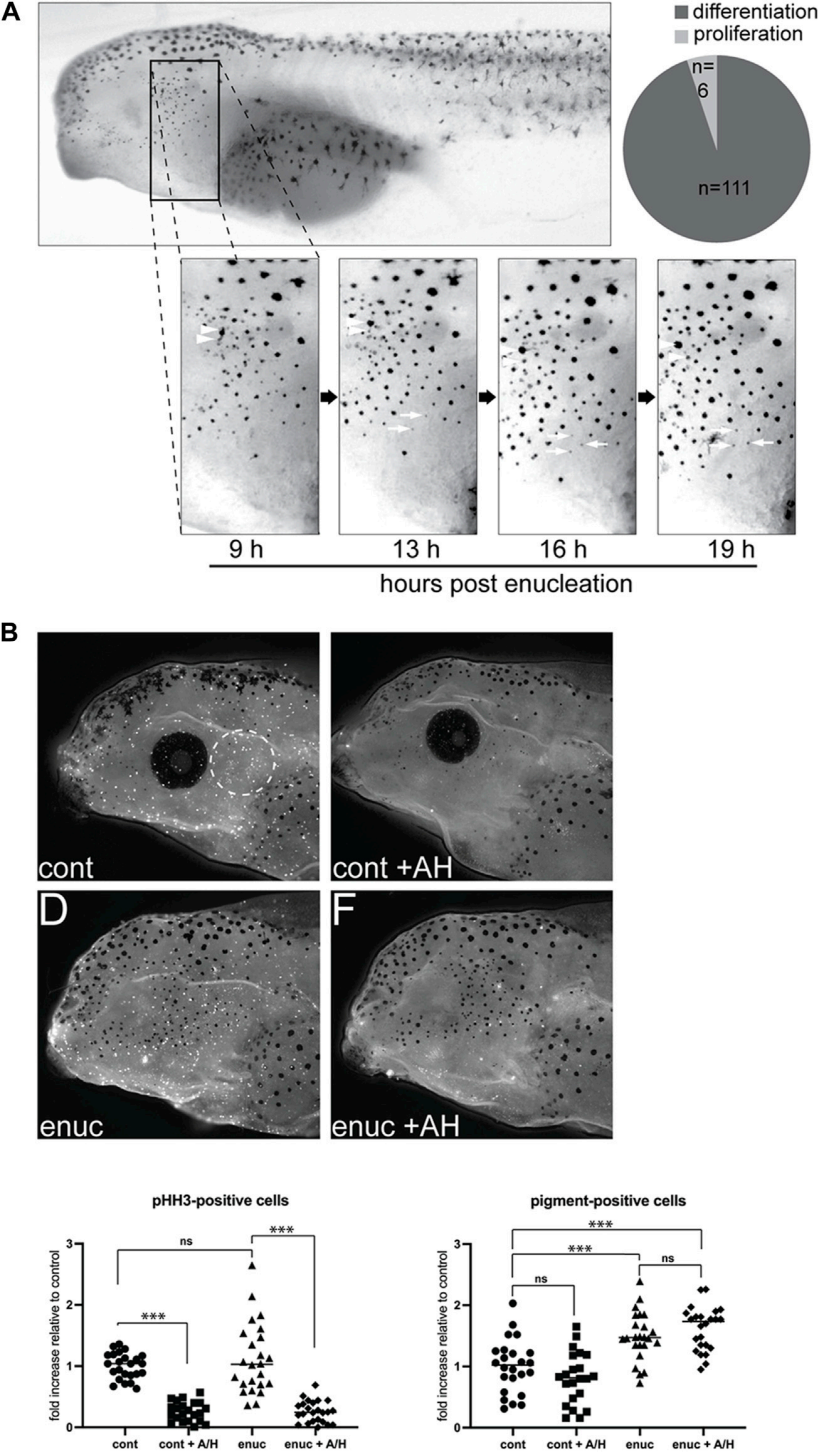
New pigmented cells emerge through *de novo* pigment production, not migration or proliferation. **(A)** To track the emergence of new melanophores, enucleated larvae were imaged from 9–19 h post-surgery. A common zone was identified across these images using anatomical landmarks so that individual cells could be tracked over time. Newly emerged pigment cells within the zone were identified (n = 117 melanophores, *N =* 6). The majority of these (n = 111) appeared *de novo* as a faint grey dot that darkened over time (white arrows). A few melanophores (n = 6) may have emerged from pigmented cell division (arrowheads). **(B)** Cell proliferation was inhibited in stage 40 control and enucleated larvae using aphidicolin-hydroxyurea (AH). The numbers of pHH3-positive and pigmented cells were assessed in the perioptic area (white dashed circle) in a blinded fashion after 24 h of AH on a white background. pHH3+ cells in the perioptic region were dramatically reduced in AH-treated larvae (pHH3-positive cells: $\bar{X}$ _*cont*_ = 1.00 ± 0.04, *n*_*cont*_ = 24; $\bar{X}$ _*cont+AH*_ = 0.26 ± 0.03, *n*_*cont+AH*_ = 22; $\bar{X}$ _*enuc*_ = 1.12 ± 0.12, *n*_*enuc*_ = 24; $\bar{X}$ _*enuc+AH*_ = 0.26 ± 0.04, *n*_*enuc+AH*_ = 24; *N* = 3; F_*3,90*_ = 45.77, *p < 0.0001*, one-way ANOVA). Whereas the enucleation-induced increase in melanophore number was not impacted (pigment-positive cells: $\bar{X}$ _*cont*_ = 1.00 ± 0.08; $\bar{X}$ _*cont+AH*_ = 0.81 ± 0.09; $\bar{X}$ _*enuc*_ = 1.49 ± 0.08; $\bar{X}$ _*enuc+AH*_ = 1.62 ± 0.07; F_*3,90*_ = 21.23, *p < 0.0001*, one-way ANOVA). ***p < 0.0001, Tukey’s test.

Results are presented as scatter plots with each replicate represented by a single point and the mean indicated by a line. The number of experiments (*N*), replicates (n), mean ( $\bar{X}$ ) and standard error (+/−) are indicated in the text. Statistical significance, which is indicated in the figures, was determined using unpaired two-tailed Student’s *t*-tests for all two-group comparisons and parametric One-Way ANOVA followed by a Tukey’s tests for all multi-group comparisons. All graphs and analyses were completed using Graph Pad Prism Software Version 9.5.1, with the exception of the pie graph in Figure 2 and the bar graphs in Figure 4D, which were created in Excel.

## 3 Results

### 3.1 Pigment cell development in the perioptic region is particularly sensitive to visual input

Previously, we found greater melanophore numbers in the skin just dorsal to the eye in larvae that experience reduced visual input, either through dark rearing or enucleation, compared with larvae reared in the light on a white background (Bertolesi, et al., 2016a). To determine if all melanophores respond similarly to light stimuli, we assessed the impact of lost visual input on melanophore number in different skin regions. Eyes were removed surgically at stage 40, when the visual system is considered functional (Chien et al., 1993; Tao et al., 2001; Bertolesi et al., 2014), and pigmentation examined 24 h later at stage 42. Melanophores were counted in a blinded fashion in a discreet region (dashed lines, Figure 1A) of four spatial zones that exhibit distinct melanophore morphology and distribution: the perioptic zone posterior to the eye, the midline of the head, the flank, and the tail (for positioning of zones see methods section). Interestingly, we observed regional differences in the impact of enucleation on melanophore number. Within the perioptic region, melanophore numbers increased significantly relative to sham surgery-treated controls 24 h after enucleation, as we observed previously for the perioptic region dorsal to the eye upon enucleation (Bertolesi et al., 2016a). Together, these data suggest that the skin surrounding the entire eye responds robustly to changes in visual input. Enucleation, however, triggered only small increases in melanophore numbers in the flank and tail regions when compared to controls. Note, in some cases more pigment was produced per melanophore in enucleated larvae, which increased the area covered by the aggregated pigment granules. While this was seen as skin darkening, it did not result from increased pigmented cell numbers (compare flank images in control and enucleated conditions). In the midline of the head, no difference in melanophore number was found. Overall, in the absence of the eye, melanophore number increases across the larva, with the perioptic region being most affected.

Previously, we found that embryos reared on a black background over a 3-day period starting at stage 34, before the visual system is functional, exhibit more pigmentation than white background-reared embryos (Bertolesi et al., 2016a; Bertolesi et al., 2017). We wanted to determine whether physiologically relevant stimuli, such as a black background exposure, can trigger an increase in melanophore number within 24 h, and after the embryonic period. Thus, we focused on the posterior perioptic zone and compared the effect of enucleation and maintenance on a black substrate with overhead lighting for a 24 h period from stage 40 to stage 42. As described above, melanophores were counted in a blinded fashion. Changing light conditions had a significant impact on perioptic melanophore number as 24 h exposure to a black background consistently triggered a significant increase relative to larvae placed on a white background, although not quite to the degree observed after enucleation (Figure 1B).

### 3.2 Visual input does not impact melanophore migration or proliferation

The vision-dependent changes in perioptic melanophores might result from changes to the proliferation, migration, or differentiation of melanophore precursors. We first considered production and migration of new melanophores. Nascent melanoblasts arise from the neural crest (Pegoraro and Monsoro-Burq, 2013). As they differentiate, melanoblasts and pigmented melanophores migrate away from the dorsal midline, travelling in a lateral and ventral direction (Frunchak and Milos, 1990; Thomas and Erickson, 2008; Kelsh et al., 2009). To address the possibility that new melanophores in the perioptic region migrated laterally and ventrally from the dorsal midline, we performed time lapse analysis to follow the emergence of new pigment-positive cells. These experiments also allowed us to assess whether new melanophores arose through division of existing ones (Frunchak and Milos, 1990). Nine hours after enucleation, larvae were briefly anesthetized with tricaine and imaged. This process was repeated every 3–4 h until 19 h post-surgery. Note that tricaine caused rapid and reversible melanosome aggregation, which allowed easy identification and tracking of individual cells within a defined region of the perioptic area.

While a small degree of dorso-ventral movement of existing perioptic melanophores was observed over the 10 h period, we did not find a single example of a new perioptic melanophore that descended from the dorsal midline. Instead, new melanophores emerged as a faint grey spot that gradually darkened over time into a pigmented spot (Figure 2A, white arrows). Of the 117 new melanophores observed (*N* = 6), 111 of them followed this process. The remaining 6 cells emerged alongside an existing melanophore in apparent examples of pigmented melanophore cell division (Figure 2A, white arrowheads), though it was possible these cells emerged from beneath an existing melanophore, with the initial appearance of a grey spot masked by an overlying more heavily pigmented melanophore. Nonetheless, the vast majority of new pigment-positive cells in the perioptic region appear to arise through a differentiation/maturation process that commences within hours of enucleation.

To confirm that new melanophores did not arise through proliferation, we asked if inhibiting proliferation with an aphidicolin-hydroxyurea (AH) cocktail blocked the emergence of new melanophores after enucleation. Importantly, these DNA replication inhibitors block proliferation in *Xenopus* and zebrafish embryos (Harris and Hartenstein, 1991; Cechmanek and McFarlane, 2017). Counting of phosphohistone H3 (pHH3) immunolabelled cells in whole mount larvae, a marker previously used to identify proliferating cells in *X. laevis* (McKeown et al., 2013), confirmed that this treatment inhibited proliferation; pHH3+ cells were reduced 70% in AH-treated larvae compared to the vehicle-treated controls after normalizing data for each clutch to yoked controls maintained in the light on a white background (Figure 2B). Despite diminished proliferation, melanophores increased 24 h after enucleation (Figure 2B). In support, enucleation increased melanophores in the absence of more pHH3+ cells. Overall, these data argue that proliferation of melanoblasts is not what regulates vision-dependent changes in melanophore numbers.

### 3.3 Visual input regulates melanophore differentiation

Our time lapse data indicate that new melanophores emerge gradually. As such, we reasoned that differentiation may be the process affected by enucleation or black background exposure. Key markers of melanophore differentiation include *tyr*, *tyrp1*, *tyrp2*, and *pmel* expression, as they associate with the capacity to synthesize melanin and form melanosomes (D’Mello et al., 2016; Dell’Angelica, 2003; Mort et al., 2015; Thomas and Erickson, 2008). The expression of *pmel* has not previously been described in *X. laevis*, therefore we compared the embryonic time course of expression of *pmel* to that of *tyr*, *tyrp1*, and *mitf*, a transcription factor critical for melanophore differentiation and the expression of the melanin synthesis enzymes using whole mount *in situ* hybridization (WMISH). In a pattern similar to that observed for *tyr, tyrp1* and *mitf*, *pmel* was evident in a small number of pre-migratory melanoblasts along the dorsal midline of the early tailbud (st. 24) embryo (Supplementary Figure S1, insets). The expression of *tyr*, *tyrp1*, and *pmel* was maintained in the retinal pigment epithelium (RPE), and in putative melanophores and melanophore precursor cells along the dorsal midline of the head, flank, and in the skin covering the somites and tail, through tailbud stages. As reported previously, *mitf* expression wanes as melanophore differentiation proceeds; at stage 32 and 35 *mitf* was expressed robustly near the tip of the tail bud where new melanophore precursors continue to emerge, but little expression was observed in mature melanophores along the flank and tail (Supplementary Figure S1; Kumasaka et al., 2004). Importantly, the *tyrp1, mitf,* and *pmel* sense probes did not label melanophore or melanophore precursor cells in embryos up to stage 40 (Supplementary Figure S2). Of note, sense probes did result in background staining, evident as faint puncta on the head and flank in pigmented melanophores of stage 42 larvae (Supplementary Figure S2).

**FIGURE 3:**
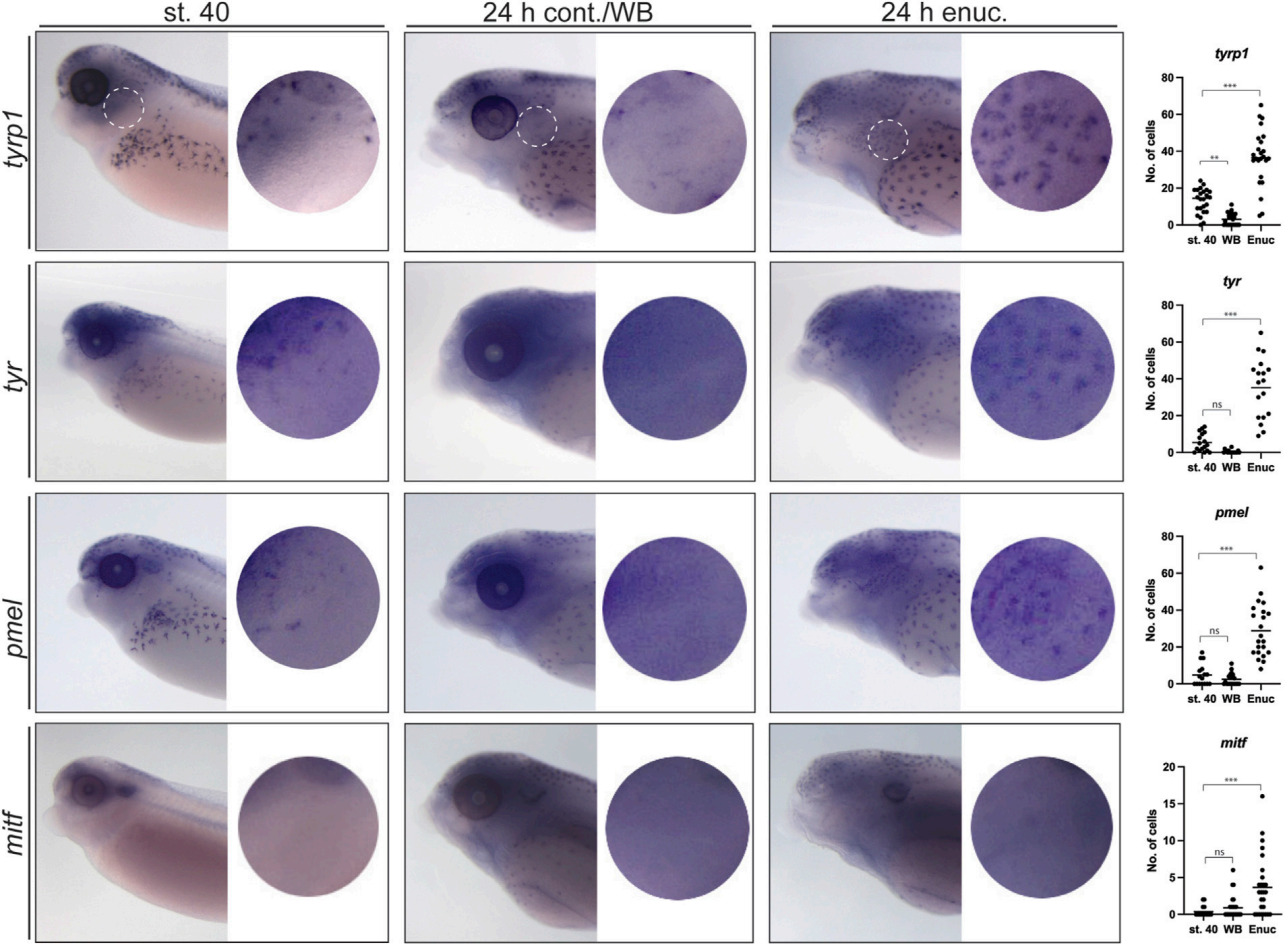
Enucleation triggers substantial changes in the expression of *tyrp1*, *tyr* and *pmel* but not *mitf* in perioptic melanophores. At stage 40, *tyrp1, tyr,* and *pmel* are expressed by a small number of perioptic melanophores as assessed by WMISH, whereas *mitf* expression is rarely visible in individual cells by this method (circular insets show perioptic region; $\bar{X}$ _*st40 tyrp1*_ = 13.04 ± 1.36, *n*_*st40 tyrp1*_ = 24, *N* = 3; $\bar{X}$ _*st40 tyr*_ = 5.41 ± 1.20, *n*_*st40 tyr*_ = 17, *N* = 2; $\bar{X}$ _*st40 pmel*_ = 4.81 ± 1.44, *n*_*st40 pmel*_ = 16, *N* = 2; $\bar{X}$ _*st40 mitf*_ = 0.38 ± 0.18, *n*_*st40 mitf*_ = 16; *N* = 2). After 24 h on a white background with illumination from above (24 h cont./WB), the number of cells expressing *tyrp*, *tyr*, and *pmel* appears reduced, while there continues to be very few *mitf* expressing cells in the perioptic region ( $\bar{X}$ _*WB tyrp1*_ = 2.96 ± 0.61, *n*_*st40 tyrp1*_ = 27; $\bar{X}$ _*WB tyr*_ = 0.50 ± 0.20, *n*_*WB tyr*_ = 18; $\bar{X}$ _*WB pmel*_ = 2.50 ± 0.67, *n*_*WB pmel*_ = 22; $\bar{X}$ _*WB mitf*_ = 0.89 ± 0.28, *n*_*WB mitf*_ = 28). In contrast, WMISH reveals a dramatic increase in *tyrp1*, *tyr*, and *pmel* expressing cells in larvae enucleated at stage 40, prior to being maintained for 24 h in the light on a white background ( $\bar{X}$ _*Enuc tyrp1*_ = 36.75 ± 3.12, *n*_*Enuc tyrp1*_ = 24; $\bar{X}$ _*Enuc tyr*_ = 35.17 ± 3.90, *n*_*Enuc tyr*_ = 18; $\bar{X}$ _*Enuc pmel*_ = 28.82 ± 3.06, *n*_*Enuc pmel*_ = 22). However, enucleation does not result in a dramatic increase in *mitf*-labeled cells in the perioptic region ( $\bar{X}$ _*Enuc mitf*_ = 3.67 ± 0.78, *n*_*Enuc mitf*_ = 27). (One-way ANOVA results: *tyrp1*:F_*2,72*_ = 81.34, *p < 0.0001*; *tyr*:F_*2,50*_ = 62.23, *p < 0.0001*; *pmel*:F_*2,57*_ = 50.88, *p < 0.0001*; *mitf*:F_*2,68*_ = 10.20, *p < 0.0001*). **p < 0.001, ***p < 0.0001, n. s., not significant, Tukey’s test.

To determine whether upregulation of *tyr*, *tyrp1,* and *pmel* mRNA underlies the visual activity-dependent emergence of perioptic melanophores, we examined the expression of these differentiation markers in the perioptic region of stage 40, 24 h post-enucleation, and 24 h white background control larvae. Larvae were treated with phenylthiourea (PTU) at the time of surgery to reduce artifactual background staining in melanophores that can occur in stage 42 larvae during the WMISH procedure (as described above). Importantly, while this timing of PTU exposure limits melanin synthesis (Karlsson et al., 2001) in new perioptic melanophores, WMISH label for melanophore differentiation markers was unaffected. A small number of labeled cells were observed in the perioptic region of stage 40 larvae stained for *tyrp1*, *tyr*, and *pmel* mRNA (Figure 3). Interestingly, a dramatic increase in *tyrp1, tyr,* and *pmel* expression was observed in cells in the perioptic region 24 h after enucleation when compared to controls (Figure 3), suggesting that upregulation of genes important for melanization, underlies the emergence of new perioptic melanophores. These findings argue that in light sensitive larvae, visual system activity controls the differentiation of melanophore precursors as a means to regulate melanophore number.

We next determined the degree to which this differentiation program reuses the program that drives pigment cell development in the embryo where *mitf* is upregulated in melanoblasts and subsequently drives the expression of melanization genes *tyr*, *tyrp1*, and *pmel* (Baxter and Pavan, 2003; Kumasaka et al., 2005; Thomas and Erickson, 2008; Braasch et al., 2009; Pegoraro and Monsoro-Burq, 2013; Mort et al., 2015; Sutton et al., 2021). Given the importance of Mitf in embryonic pigmentation, we examined the impact of enucleation on *mitf* expression. In stage 40 larvae, no *mitf*-positive cells were observed in the perioptic region (Figure 3). Surprisingly, unlike the melanization genes, only a very small increase in *mitf* expression was observed in the 24 h post-enucleation group (Figure 3). Note, the puncta visible in the dorsal head, dorsal midline of the body, and along the flank, were artefactual and also observed with the sense probe (Supplementary Figure S2). Collectively, these data suggest that post-sensory melanophore differentiation in the perioptic area differs from embryonic differentiation, where *tyrp1*, *tyr*, and *pmel* upregulation occurs without an obvious underlying increase in *mitf* mRNA.

**FIGURE 4:**
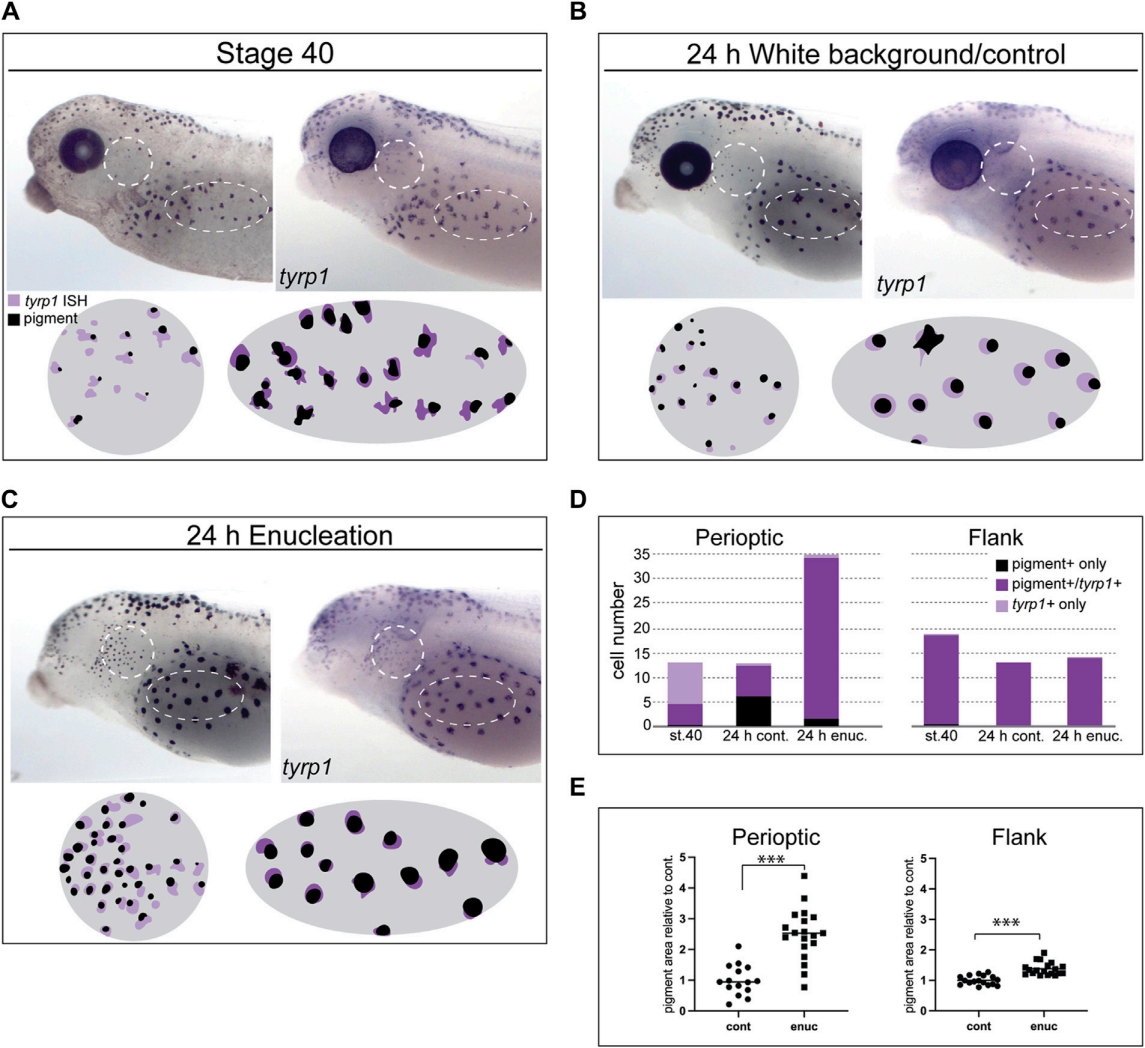
Loss of visual input increases *tyrp1* expression and perioptic melanophore differentiation. **(A–C)** *tyrp1* mRNA compared to pigmentation for each larva by overlaying WMISH and pigment aggregate images (illustrated in circle and oval schematics for perioptic and flank areas, respectively) at stage 40 **(A)**, after 24 h on a white background **(B)**, and 24 h post-enucleation **(C)**. **(D)** Average number of pigmented + only, pigmented+/*tyrp1*+, and *tyrp1*+ only cells for each condition shown in A-C for the perioptic area and flank. White dashed circle (perioptic region) and oval (flank) indicate analysis regions. (Perioptic-st.40: $\bar{X}$ _*tyrp1+only*_ = 8.3 ± 1.0, $\bar{X}$ _*tyrp1+/pig+*_ = 4.4 ± 0.5, $\bar{X}$ _*pig+only*_ = 0.4 ± 0.3, *n*_*st40*_ = 26; perioptic-cont: $\bar{X}$ _*tyrp1+only*_ = 0.4 ± 0.1, $\bar{X}$ _*tyrp1+/pig+*_ = 6.3 ± 1.7, $\bar{X}$ _*pig+only*_ = 6.2 ± 1.2, *n*_*cont/WB*_ = 25; perioptic-enuc: $\bar{X}$ _*tyrp1+only*_ = 0.6 ± 0.2, $\bar{X}$ _*tyrp1+/pig+*_ = 32.6 ± 1.5, $\bar{X}$ _*pig+only*_ = 1.6 ± 0.5, *n*_*enuc*_ = 25; *N* = 3) (Flank-st.40: $\bar{X}$ _*tyrp1+only*_ = 0.1 ± 0.1, $\bar{X}$ _*tyrp1+/pig+*_ = 18.2 ± 1.4, $\bar{X}$ _*pig+only*_ = 0.1 ± 0.1, *n*_*st40*_ = 16; flank-cont: $\bar{X}$ _*tyrp1+only*_ = 0.0 ± 0.0, $\bar{X}$ _*tyrp1+/pig+*_ = 12.8 ± 0.6, $\bar{X}$ _*pig+only*_ = 0.0 ± 0.0, *n*_*cont/WB*_ = 25; flank-enuc: $\bar{X}$ _*tyrp1+only*_ = 0.0 ± 0.0, $\bar{X}$ _*tyrp1+/pig+*_ = 13.7 ± 0.8, $\bar{X}$ _*pig+only*_ = 0.1 ± 0.1; *n*_*enuc*_ = 25; *N* = 2) **(E)** Average pigment aggregate area (perioptic: $\bar{X}$ _*cnt*_ = 1.00 ± 0.13, *n*_*cnt*_ = 15; $\bar{X}$ _*enuc*_ = 2.49 ± 0.19, *n*_*enuc*_ = 19; flank: $\bar{X}$ _*cnt*_ = 1.00 ± 0.04; $\bar{X}$ _*enuc*_ = 1.38 ± 0.05; *N* = 2). ***p < 0.0001, unpaired two-tailed Student’s *t*-test.

To further characterize the larval melanophore differentiation process, we tracked differentiation by directly comparing the presence of pigmentation synthesis enzymes with pigmentation at an individual melanophore level in enucleated larvae by aligning patterns of pigment and *tyrp1* WMISH label. We chose the *tyrp1* probe for this analysis as it results in a dark, crisp stain. At stage 40, many perioptic cells were both pigmented and *tyrp1*+ (Figure 4A). This result was expected as Tyrp1 contributes to melanin production (D’Mello et al., 2016). A second population of cells were *tyrp1*+ but pigment negative (*tyrp1*+ only), and were likely captured as these cells increased *tyrp1* transcription in preparation for pigment production. We quantified these distinct cell populations and determined that three populations of cells existed at stage 40: a small population of pigmented yet *tyrp1*-cells (pigment + only), and two larger populations of unpigmented and pigmented *tyrp1*+ cells (Figures 4A, D). The presence of unpigmented *tyrp1*+ cells in the perioptic region aligns with our time lapse data that showed new pigmented cells emerge through a gradual increase in pigmentation. Overall, these data suggest the perioptic cells exhibit a spectrum of differentiation at stage 40, with some cells having differentiated and others only partially mature and lacking pigment. Note that potentially a fourth population exists in the perioptic region, which we were unable to account for in this analysis: undifferentiated melanophores that lack pigment and *tyrp1* expression. The situation in the perioptic area was in contrast to the flank at this stage, where virtually all the cells were pigmented *tyrp1*+ melanophores (Figures 4A, D). This scenario also appeared true of the dorsal and tail regions, although we did not quantify these melanophore populations.

With both ongoing larval development and enucleation, the distribution amongst the perioptic melanophore populations switched, though in opposite manners. With larval development, *tyrp1*+ cell numbers were reduced in control embryos developing for 24 h on a white background (Figures 4B, D), with an increase in pigmented cells lacking *tyrp1* expression. Potentially, these cells reflect a de-differentiation process towards an immature state, where the cells stop producing the pigment synthesis enzymes, yet still retain some previously produced pigment granules. A subset of pigmented cells continued to express *tyrp1*. In contrast, in the flank all cells were both pigmented and *tyrp1+*, despite exposure to 24 h of light on a white background (Figures 4B, D), arguing that cells maintain their mature state in this region. Enucleation caused a dramatic increase in pigmented *tyrp1*+ cells in the perioptic region, but had little or no effect on the pigmented *tyrp1+* flank melanophores (Figures 4C, D). These data argue that visual input controls a final step in perioptic melanophore differentiation, with a population of melanophores remaining in an undifferentiated state, ready to quickly alter *tyrp1* expression in response to persistent changes in visual activity. This flexibility does not appear to be a feature of flank melanophores.

When compared to control larvae, the size of aggregated melanosome patches appeared larger in enucleated larvae in both the flank and perioptic areas (compare melanophores in Figures 4B, C), likely reflecting a real difference in pigment levels. To quantify the relative change in pigment levels, the average area of pigment aggregates within the analysis zone of each embryo was measured and normalized to controls to account for variations in baseline pigment production between clutches. Perioptic melanophores exhibited a 2.5-fold increase in aggregate size, while flank melanophores showed a 1.4-fold increase (Figure 4E). Thus, pigment production was increased in both regions, suggesting visual input controls *tyrp1* expression and pigment production across melanophores.

### 3.4 Undifferentiated melanophores are maintained as larvae mature

**FIGURE 5:**
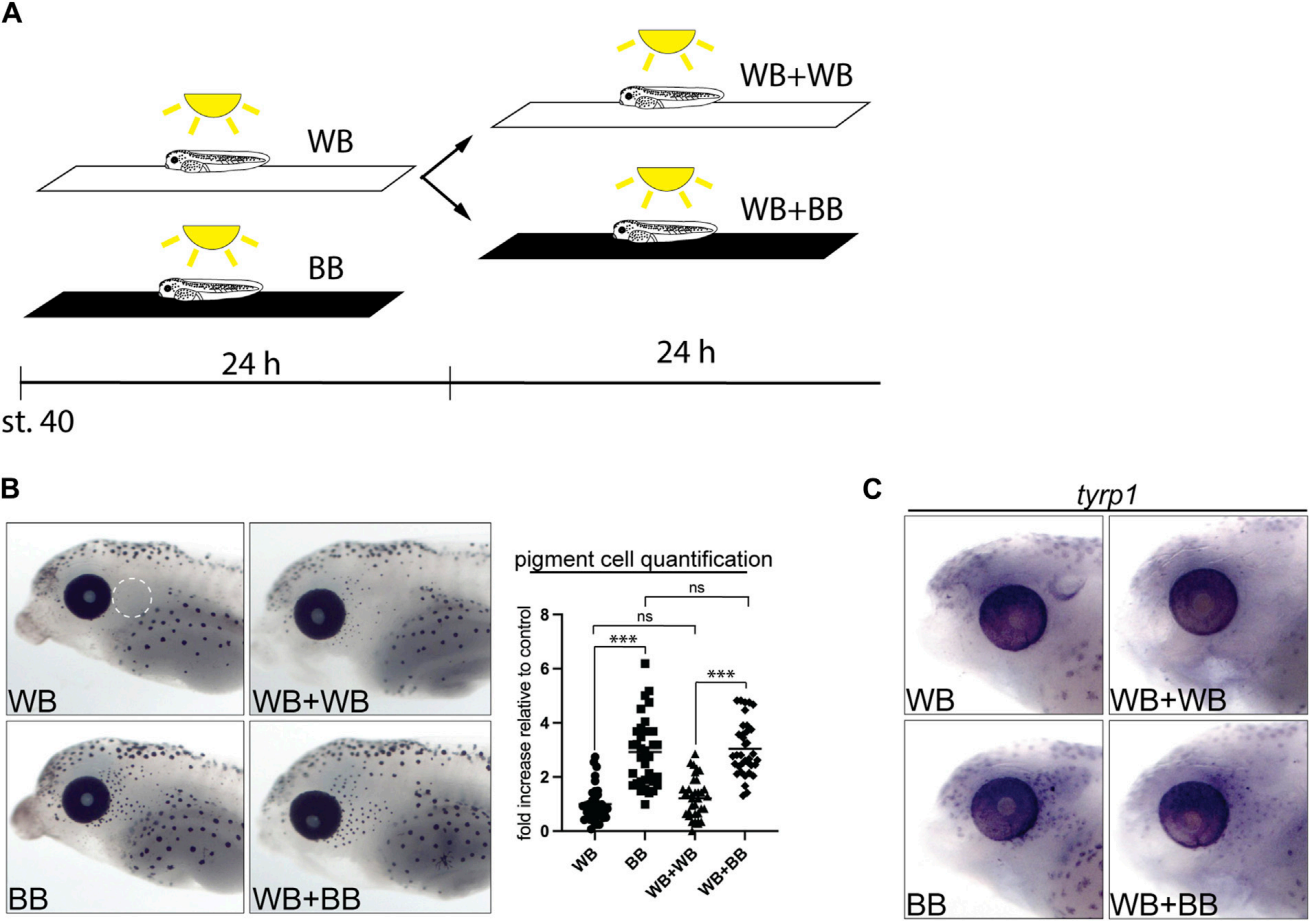
Undifferentiated melanophores are maintained through larval stages. **(A)** Stage 40 larvae were exposed to different backgrounds while being illuminated from above for 24–48 h. **(B)** Larvae were maintain for 24 h on a white background (WB), for 48 h on a white background (WB + WB), for 24 h on a black background (BB), and for 24 h on a white background followed by 24 h on a black background (WB + BB). Perioptic melanophores were quantified (dashed circle in A; $\bar{X}$ _*WB*_ = 1.00 ± 0.11, *n*_*WB*_ = 34; $\bar{X}$ _*BB*_ = 2.92 ± 0.21, *n*_*BB*_ = 35; $\bar{X}$ _*WB+WB*_ = 1.22 ± 0.13, *n*_*WB+WB*_ = 35; $\bar{X}$ _*WB+BB*_ = 3.04 ± 0.17, *n*_*WB+BB*_ = 36; *N* = 4; F_*3, 136*_ = 46.11, *p < 0.0001*, one-way ANOVA). **(C)** WMISH for *tyrp1* in the perioptic area for the four conditions. ***p < 0.0001, Tukey’s test.

We next asked whether beyond stage 40 the quiescent, unpigmented melanophore population remained available to generate new pigmented melanophores upon changes in environmental light. Larvae were first grown on a white background for 24 h to suppress the maturation of perioptic melanophores (WB), and then for the subsequent 24 h either left on the white background (WB + WB) or switched to a black background (WB + BB; Figure 5A). When larvae were maintained on a white background for 24 or 48 h, pigment cell numbers remained low (Figure 5B). When exposure to the black background was delayed by 24 h (WB + BB), an increase in pigmented cells was observed, comparable to that seen after 24 h exposure to a black background at stage 40 (BB, Figure 5B). Blinded quantification of melanophore number confirmed these observations. To determine whether *tyrp1* regulation continued to underlie perioptic melanophore maturation at these later stages, we examined *tyrp1* mRNA by WMISH in the WB + WB and WB + BB conditions (Figure 5C). Importantly, as we observed after enucleation, the 24 h black background exposure beginning at stage 40 produced an elevation in *tyrp1* expression in perioptic cells (compare WB and BB in Figure 5C). This increase in *tyrp1*+ cells was also observed in larvae exposed to white background for 24 h followed by black background (compare WB + WB and WB + BB in Figure 5C). Together, these data indicate that the undifferentiated population of melanophores is still present a day later than stage 40, arguing that this immature population is maintained for a period of development.

### 3.5 Neural activity from the eye and a melatonin signal regulate melanophore number

**FIGURE 6:**
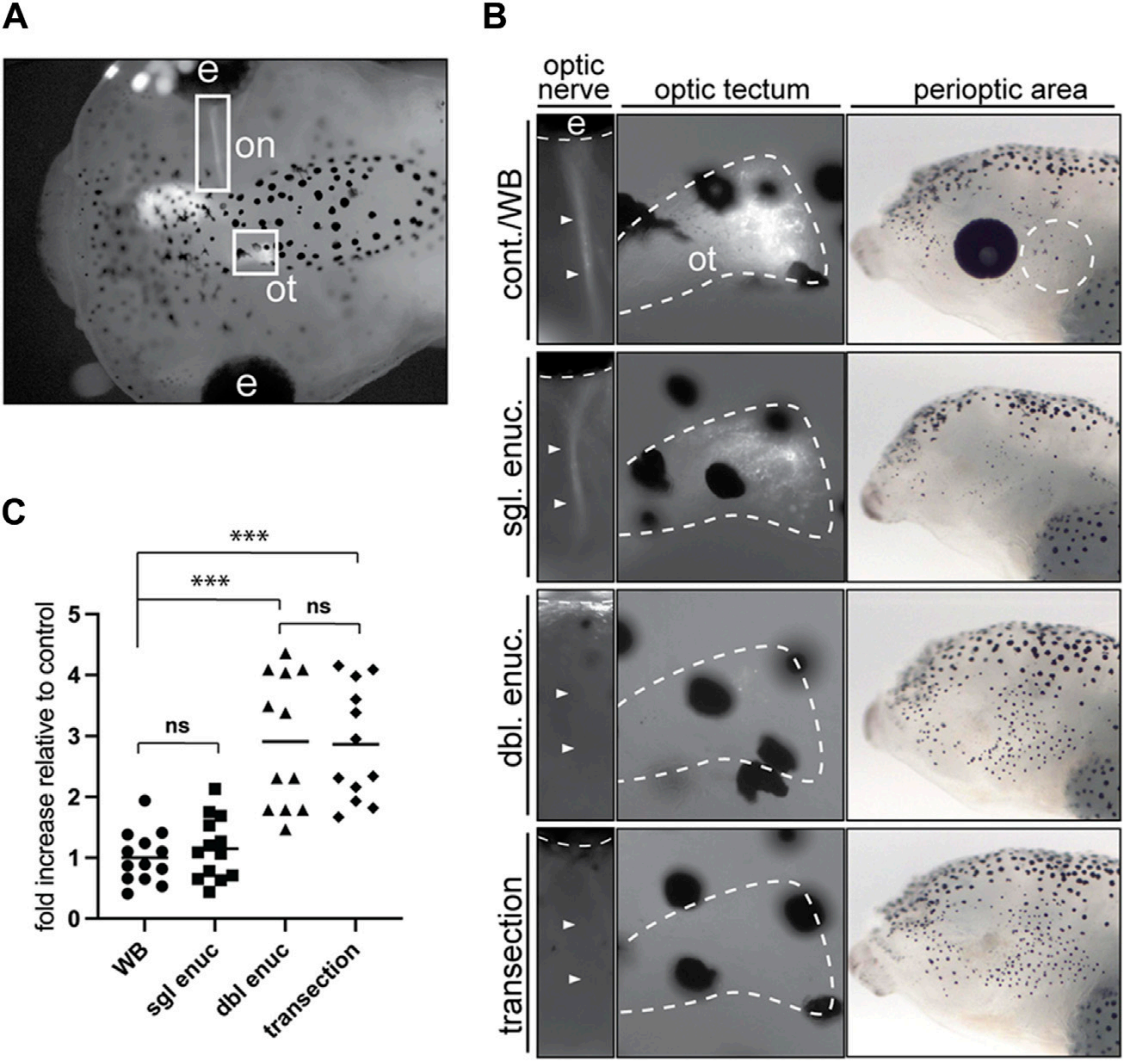
Optic nerve transection mimics the enucleation-induced increase in melanophore number. **(A)** Larvae with a unilaterally GFP-labeled optic nerve (on) and optic tectum (ot) underwent sham surgery (cont./WB), single enucleation (sgl. enuc.), double enucleation (dbl. enuc.), or single enucleation with transection of the remaining optic nerve (transection). **(B)** Images of representative larvae from each surgical condition. The first panel shows presence or absence of GFP-positive optic nerve (arrowheads), second panel shows the presence or absence of GFP-positive retinal ganglion cell axon terminals in the optic tectum (white dashed line), and third panel shows lateral brightfield view of perioptic region for each condition. **(C)** 24 h after enucleation and/or optic nerve transection or sham surgery, melanophore numbers in the perioptic region (white dashed circle in B, third panel) were compared; $\bar{X}$ _*cont*_ = 1.00 ± 0.12, *n*_*cont*_ = 13; $\bar{X}$ _*sgl enuc*_ = 1.15 ± 0.14, *n*_*sgl enuc*_ = 12; $\bar{X}$ _*dbl enuc*_ = 2.91 ± 0.32; *n*_*dbl enuc*_ = 12; $\bar{X}$ _*trans*_ = 2.87 ± 0.94, *n*_*trans*_ = 12; *N* = 2; F_*3,46*_ = 23.06, *p < 0.0001*, one-way ANOVA). ***p < 0.0001, n. s., not significant, Tukey’s test.

We next addressed the nature of the eye signal. Given that the perioptic melanophore populations is the most impacted by enucleation and black-background exposure, we reasoned the eye may act in a paracrine fashion to regulate perioptic melanophore differentiation, analogous to how the eye organizes vertebrate craniofacial morphogenesis through the release of retinoic acid and sonic hedgehog (Kish et al., 2011). Alternatively, neural signals from the eye to the brain may control pigment cell maturation. To distinguish between neural and paracrine mechanisms, we compared larval perioptic melanophore numbers 24 h after enucleation or optic nerve transection. We eye electroporated CS2-GFP at stage 28 to confirm in each stage 42 embryo the success of transection by the absence of a GFP-labeled optic tract (Figure 6A). To avoid having to both successfully electroporate and subsequently transect the optic nerves of both eyes in each larva, we took advantage of the observation that removal of one eye has no impact on melanophore number (Bertolesi, et al., 2016a). Thus, larvae with one well electroporated eye had the other eye removed. Unilaterally enucleated larvae then underwent optic nerve transection of the remaining eye. Note that if the paracrine mechanism was key, the single eye remaining could maintain the low melanophore number. Perioptic melanophores were counted for all embryos after 24 h on a white background in the light. Images for representative larvae, showing the presence or absence of the fluorescent optic nerve fibers (first panel) and their tectal terminals (second panel) as well as a lateral brightfield view of the head (third panel), are provided for each condition (Figure 6B).

As expected, unilaterally enucleated larvae (sgl enuc) were indistinguishable from controls in pigment-positive cell numbers (Figure 6C). However, severing the remaining optic nerve (transection) in single-enucleated larvae resulted in an almost three-fold increase in melanophores relative to control and single enucleated larvae. Furthermore, transected larvae were indistinguishable from bilaterally (double) enucleated larvae, suggesting that the eye does not act in a paracrine manner to regulate melanophore number. Instead, these data argue the eye sends a neural signal via the optic nerve to suppress perioptic melanophore numbers.

**FIGURE 7:**
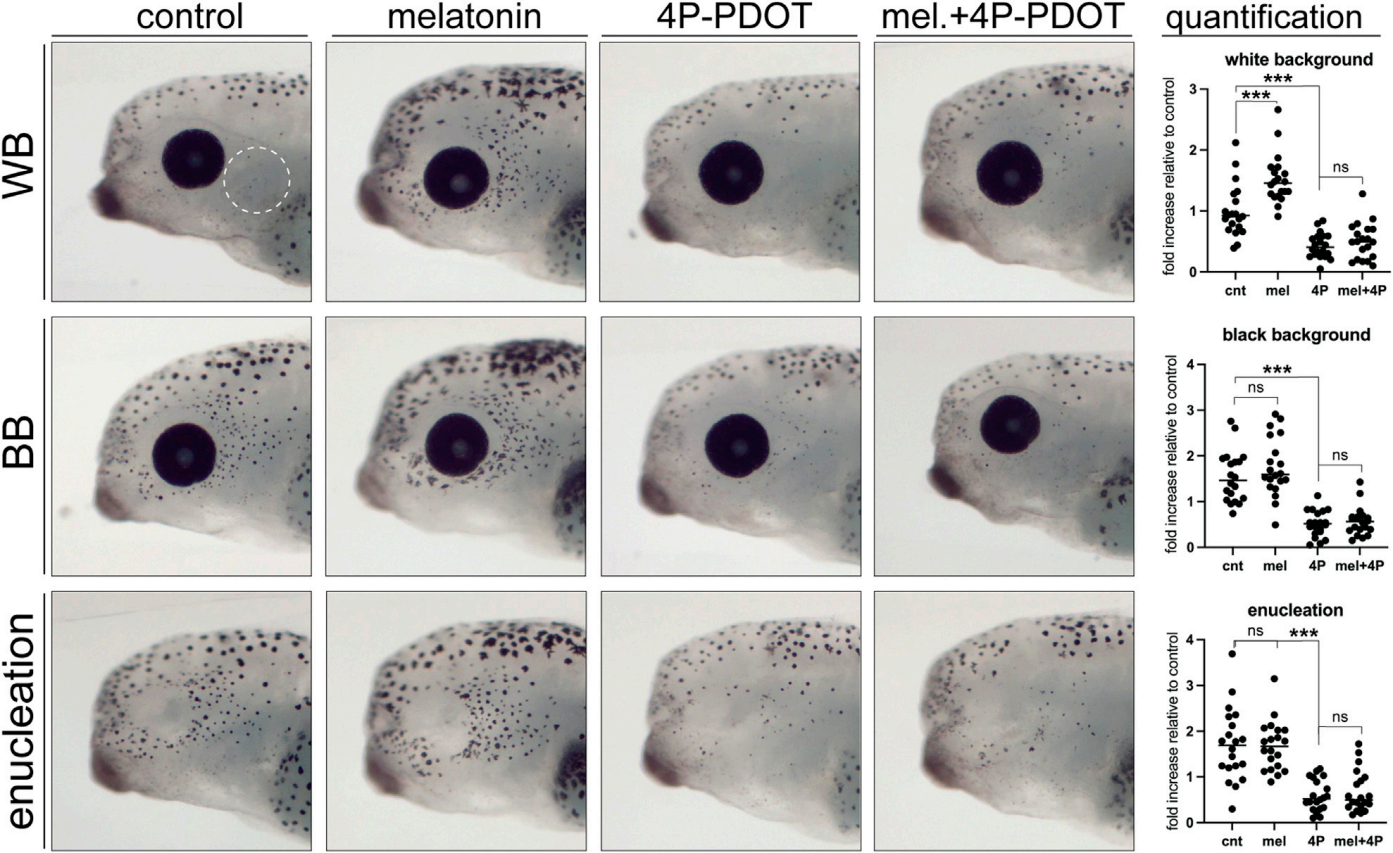
Melatonin signalling is necessary and sufficient to induce perioptic melanophore differentiation. Stage 40 larvae underwent sham surgery and were maintained in the light on a white background or black background, or were enucleated. Larvae from each condition were exposed to control, melatonin, melatonin receptor antagonist (4P-PDOT), or melatonin and 4P-PDOT solutions for a 24-h period. Melanophore numbers in the perioptic region (white dashed circle) were compared; White background: $\bar{X}$ _*cnt*_ = 1.00 ± 0.4, *n*_*cnt*_ = 20; $\bar{X}$ _*mel*_ = 1.51 ± 0.4, *n*_*mel*_ = 20; $\bar{X}$ _*4PPDOT*_ = 0.43 ± 0.2, *n*_*4PPDOT*_ = 20; $\bar{X}$ _*mel+4p*_ = 0.50 ± 0.30, *n*_*mel+4p*_ = 20; *N* = 2; F_*3,79*_ = 42.34, *p < 0.0001*, one-way ANOVA; Black background: $\bar{X}$ _*cnt*_ = 1.54 ± 0.5, *n*_*cnt*_ = 20; $\bar{X}$ _*mel*_ = 1.76 ± 0.6, *n*_*mel*_ = 20; $\bar{X}$ _*4PPDOT*_ = 0.51 ± 0.3, *n*_*4PPDOT*_ = 20; $\bar{X}$ _*mel+4p*_ = 0.56 ± 0.3, *n*_*mel+4p*_ = 20; *N* = 2; F_*3,79*_ = 37.13, *p < 0.0001*, one-way ANOVA; Enucleation: $\bar{X}$ _*cnt*_ = 1.70 ± 0.8, *n*_*cnt*_ = 20; $\bar{X}$ _*mel*_ = 1.68 ± 0.5, *n*_*mel*_ = 20; $\bar{X}$ _*4PPDOT*_ = 0.59 ± 0.3, *n*_*4PPDOT*_ = 20; $\bar{X}$ _*mel+4p*_ = 0.67 ± 0.5, *n*_*mel+4p*_ = 20; *N* = 2; F_*3,79*_ = 23.90, *p < 0.0001*, one-way ANOVA.***p < 0.0001, Tukey’s test.

Our previous research showed that long-term changes in melatonin signaling over the embryonic period mimics the effect of enucleation and triggers an increase in the number of melanophores in the lateral head region (Bertolesi et al., 2016a). To determine whether melatonin signaling also regulates rapid changes in melanophore differentiation in post-embryonic larvae, we exposed stage 40 larvae in white background, black background, and enucleation conditions to a control or melatonin (100 μM) solution for 24 h. Larvae were then fixed, imaged, and perioptic melanophores were counted in a blinded fashion. In larvae maintained on a white background, 24 h melatonin exposure resulted in a significant increase in perioptic melanophores that was indistinguishable from that triggered by black background exposure or enucleation (Figure 7; compare WB + melatonin to BB and enucleated controls). Furthermore, melatonin did not result in a further increase in perioptic melanophores in enucleated larvae or larvae maintained on a black background. Significantly, 24 h exposure to a 50 μM solution of 4P-PDOT, an established melatonin receptor inhibitor in *X. laevis* (Prada and Udin, 2005), prevented the black background exposure and enucleation induced increase in perioptic pigment cell number, suggesting these interventions rely on a melatonin signal to promote melanophore differentiation (Figure 7). Exposure to 4P-PDOT also resulted in a small, but significant decrease in pigment cell number in control larvae (Figure 7), which argues that a baseline level of melatonin may initiate the development of the few perioptic melanophores that arise under white background conditions. Importantly, treatment with 4P-PDOT abolished the melatonin-induced increase in melanophores, arguing that the receptor antagonist is indeed blocking the melatonin receptor. Together, these data argue that a neural activity in the retina regulates a melatonin signal, which ultimately controls melanophore differentiation.

## 4 Discussion

We demonstrate that visual sensory input regulates the differentiation of neural-crest derived pigment cells. When visual input in *X. laevis* larvae is altered at stage 40, through enucleation or by placing larvae on a black background, melanophore number increases dramatically and selectively in the perioptic region. The melanophores appear to emerge through differentiation of unpigmented cells already present in the perioptic region. We propose that the upregulation of key melanization genes, *tyr*, *tyrp1,* and *pmel* by reduced neural activity in the optic nerve is the critical driver of melanophore differentiation. The number of *tyrp1*-expressing cells decreases if larvae are maintained in the light on a white background for 24 h from stage 40, suggesting that melanophores can return to an immature state. These undifferentiated melanophore precursors are likely maintained through to metamorphic stages within the perioptic region as they still activate when we expose larvae to a black background at a later developmental time point. Finally, melatonin appears to be a key signal required to promote melanophore maturation. Overall, our data suggest visual system activity controls differentiation to link melanophore development to environmental conditions.

**FIGURE 8:**
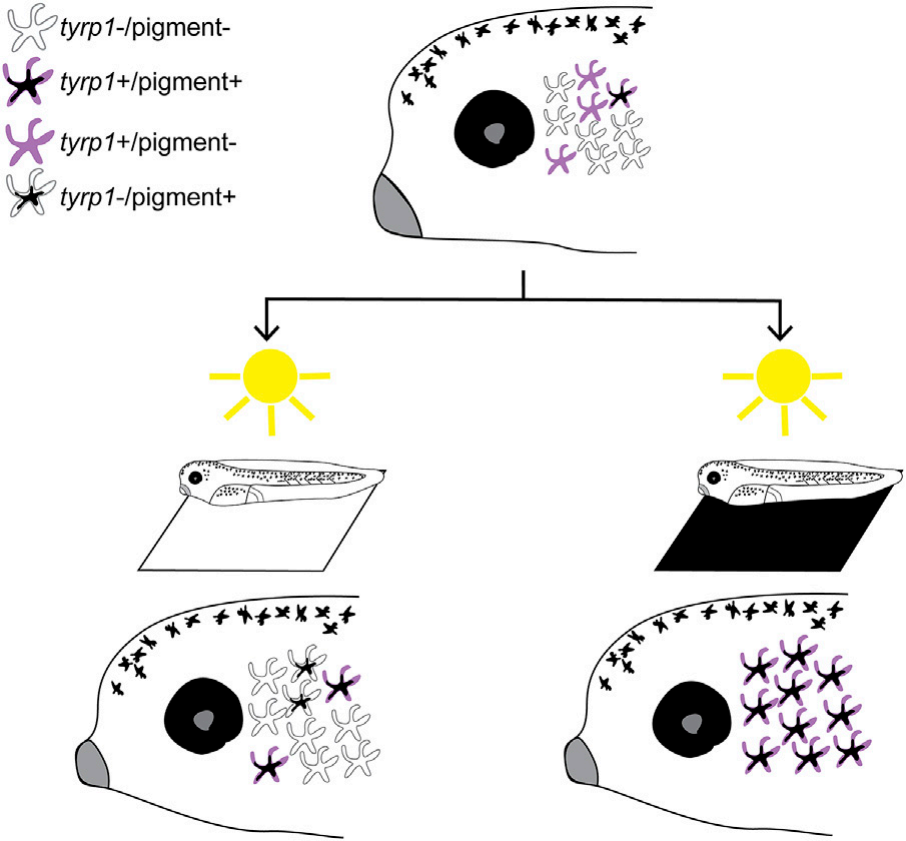
Model of vision-mediated melanophore differentiation. At stage 40 (top), as the visual system becomes functional, four related perioptic melanophores exist: 1) differentiated pigment+/*tyrp1*+, 2) differentiating *tyrp1*+/pigment-, 3) immature *tyrp1*-/pigment-awaiting a signal to differentiate, and 4) de-differentiating *tyrp1*-/pigment+. Light on a white background (bottom left) produces a small increase in pigmented cells, but *tyrp1*+ cells (pigmented and non-pigmented) decrease. Light on a black background (or 24 h enucleation; bottom right) dramatically increases pigmented and *tyrp1*+ melanophores. A black background drives differentiation of the *tyrp1*-/pigment-population into mature pigmented melanophores.

When visually active larvae are placed on a black background or undergo enucleation, we observed that new melanophores appear over the next 24 h. Time lapse analysis and our proliferation inhibition experiments reveal that the new perioptic melanophores do not emerge through proliferation of existing melanophores, nor do they migrate ventrally from the dorsal midline, the primary process for early melanophore development from neural crest cells (Thomas and Erickson, 2008; Mort et al., 2015). Instead, we propose a model where the perioptic region is populated by transparent cells whose differentiation into mature, pigment-synthesizing, melanophores is regulated by visual input (Figure 8). This mechanism becomes active when the visual circuits become functional around stage 40. The perioptic region is dynamic, containing multiple populations of melanophores that can transition between one another. These populations include: 1) *tyrp1+*/pigmented fully differentiated melanophores, 2) *tyrp1*+/unpigmented cells, progressing towards a mature differentiated state, 3) a small population of *tyrp1*-/pigmented cells, possibly transitioning towards an immature state, and likely 4) a *tyrp1*-/unpigmented population. Based on the speed at which they acquire their mature phenotype, and the observation that a significant increase in *mitf*, a key transcription factor required for melanophore differentiation, is not required for their maturation, we propose that the *tyrp1*-/unpigmented cells are arrested in their development awaiting the appropriate visual input. Distinct visual environments, such as a black background or removing visual input through enucleation, push these undifferentiated melanophores to reveal themselves through the upregulation of *tyr*, *tyrp1,* and *pmel* mRNA, a critical step in the pathway to differentiation and maturation. Conversely, maintaining larvae for 24 h in the light on a white background leads to a reduction in the number of *tyrp1*+ cells, suggesting that this light environment suppresses differentiation and drives mature melanophores back towards an immature state. Interestingly, mature melanophores can cease melanin production and transdifferentiate into leucophores in adult zebrafish, suggesting differentiation is not a terminal state in some melanophores (Lewis et al., 2019).

Intriguingly, we were unable to detect upregulation of *mitf* in enucleated larvae, or larvae exposed to a black background. MITF is a master regulator of melanocyte development: it regulates the expression of *tyr, tyrp1, tyrp2*, and *pmel* in a wide range of species, and multiple signaling pathways that lead to an increase in pigmentation and melanocyte number converge on MITF (Baxter and Pavan, 2003; D’Mello et al., 2016; Kawakami and Fisher, 2017; Mort et al., 2015; Vachtenheim and Borovanský, 2010). In *X. laevis* tadpoles, *mitf* overexpression increases melanophore numbers, and an increase in *mitf* expression clearly precedes that of the melanin synthesis enzymes in melanophores across the body (Supplementary Figure S1) (Kumasaka et al., 2004; Kumasaka et al., 2005). Yet, we failed to detect *mitf* expression in perioptic cells after stage 40 enucleation or exposure to a black background. Possibly, the Mitf protein is already present in sufficient quantities and undergoes post-translational activation through phosphorylation, a regulatory mechanism that has been observed in cultured osteoclast-like cell differentiation (Mansky et al., 2002). Alternatively, *mitf* expression might be upregulated minimally, or only transiently, in these cells and we missed capturing a change in expression by ISH. In *Oreolalax rhodostigmatus,* RNA-seq data suggest that a light-induced increase in pigment production associates with *mitf* upregulation, with higher levels of expression 4 hours after the onset of light exposure than 30 h, suggesting that expression turns on early and then wanes even as pigmentation proceeds (Zhu et al., 2018). The complete lack of *mitf*-expressing cells in *Xenopus* larvae after stage 40, however, speaks against this possibility, particularly given the strong *mitf* WMISH signal that precedes melanization gene expression in embryonic melanophores. It is also possible, *tyr*, *tyrp1* and *pmel* expression is not controlled by Mitf in the late emerging melanophores. Interestingly, MITF-independent regulation of melanization gene expression is observed in some melanoma cells. For instance, *TYRP1* transcript levels fluctuate independently of *MITF* in patient-derived melanoma cells and in the SK-MEL-19 human melanoma cell line (Fang and Setaluri, 1999; Hartman and Czyz, 2020). Whether or not MITF plays a role in visual input-controlled melanophore differentiation remains to be determined.

It is worth noting that the development of *perioptic* melanophores is uniquely sensitive to the light environment, suggesting that melanophores in the lateral head region represent a distinct subpopulation. Enucleation induces a dramatic increase in the number of *tyrp1*+/pigmented cells in the perioptic region, whereas melanophore populations elsewhere on the body exhibit little or no increase in pigmentation. Melanophores across the body could sit on a differentiation continuum stretching from unpigmented to heavily pigmented. In this scenario, perioptic melanophores would be at one end of the spectrum as mainly unpigmented, while flank melanophores would exist at the other end of the spectrum as largely pigmented at larval stages. Enucleation-driven upregulation of *tyrp1* and subsequent pigment production would bring more perioptic than flank melanophores beyond the threshold of being visible based on pigmentation. When larvae are maintained on a white background, however, *tyr, tyrp1,* and *pmel* mRNAs are maintained in flank but not perioptic melanophores. While it is possible that similar mechanisms are at play in the increase in pigmentation in perioptic and flank melanophores, the degree to which the perioptic melanophores are impacted places them in a unique category where their differentiation is intimately linked to visual system function. Based on the expression data we suggest the existence of melanophore subpopulations that have distinct localization across the body. In agreement, melanophores in different regions of the body emerge with distinct developmental patterns and possess unique morphological characteristics, including distinct sizes and pigmentation levels (Frunchak and Milos, 1990). Why would the perioptic melanophores have this unique light-sensitivity? Possibly it relates to the position and role of perioptic tissue. As *X. laevis* mature through this larval period they shift from floating on their sides to swimming upright, and the perioptic zone comes to make up a large part of the surface area of the tadpoles viewed from a dorsal vantage point. The natural signal to promote perioptic melanophore differentiation and darken the skin around the eye is a black background. The camouflage advantage by darkening the head of the larvae when on a black background may be the feature that accounts for the connection between the development of perioptic melanophores and the light environment. A deeper understanding of melanophore differentiation will be essential in understanding how melanophore subpopulations support *X. laevis* survival.

Our data suggest that melatonin positively regulates larval melanophore differentiation, apparently in a neural activity-dependent manner: melatonin increases pigment cell numbers in white background-treated larvae, while the melatonin receptor inhibitor blocks the increase in pigment cell numbers induced by a black background or enucleation. This aligns with our previous findings showing that long term (4 d) melatonin exposure from the early tailbud to larval stage triggers an increase in melanophore number in the dorsal lateral head (Bertolesi et al., 2016a). The synthesis and secretion of melatonin in response to changes in the light environment is a highly conserved feature of the neuroendocrine pineal gland (Sapède and Cau, 2013). Surprisingly, our data argue that the melatonin signal regulating larval perioptic melanophore differentiation does not originate from the pineal complex. First, we showed previously that pinealectomy does not impact perioptic melanophore number in both control and enucleated embryos (Bertolesi et al., 2016a). In addition, because light is present and illuminates the larvae from above in both the enucleation and black background scenarios, pigment cell numbers would be expected to decrease and not increase with light-mediated suppression of pineal melatonin release.

Instead, we propose that melatonin participates in a neurally active circuit initiating in the eye, as supported by our optic nerve transection data. Our distinct environmental scenarios provide hints as to features of melatonin release. We propose that with a white background, melatonin secretion is minimal, as melatonin treatment increases pigment cell numbers. Note that there is some baseline activity of the pathway, as the melatonin receptor inhibitor (4P-PDOT) further reduces pigment cell numbers in WB-exposed larvae. In contrast, a black background causes maximal melatonin release, and pigment cell numbers increase. Further application of melatonin has no impact, and melatonin receptor inhibition eliminates this key differentiation signal. Potentially, melatonin is released within the eye to impact retinal activity, in that multiple retinal cell types, including the retinal pigmented epithelium and retinal ganglion cells (RGCs), can synthesize melatonin (Acuña-Castroviejo et al., 2014). Melatonin could then influence the activity of retinal circuits, with the enucleation and black background data arguing that this melatonin would ultimately inhibit optic nerve activity; a black background would enhance retinal melatonin levels and optic nerve inhibition, while eye enucleation would simply mimic the black background scenario by eliminating any optic nerve input to the brain. Alternatively, light-mediated changes in eye neural activity may control melatonin release in the brain; either directly by RGC axon terminals or via RGC-mediated modulation of melatonin release from brain cells downstream in the circuit. The former possibility is somewhat discordant with the enucleation data, as one would expect enucleation to remove the melatonin source and reduce pigment cell numbers, though it is possible that the severing of RGC axons results in dysregulated (or injury-induced) release of melatonin from the RGC nerve terminal. Another possibility is that black background exposure and eye enucleation could both reduce an optic nerve-dependent inhibitory influence on melatonin release from extrapineal brain cells. In support, certain brain cells can synthesize melatonin, and melatonin receptor expression is widespread in the brain (Acuña-Castroviejo et al., 2014). Future experiments can identify the source of melatonin, but regardless, melatonin appears to be a critical regulator of light-dependent regulation of melanophore differentiation.

Our model proposes a population of undifferentiated melanophores is maintained through the larval period in order to provide the embryo with the capacity to rapidly respond to changes in the environment by triggering differentiation. This developmental mechanism is distinct from many examples of experience-dependent regulation of development, where cell morphogenesis is the primary process impacted. Examples of the impact of external cues on cell morphology center around the nervous system and include studies of early environmental deprivation or enrichment, which influences dendritic branching, synaptogenesis and connectivity in many brain regions. These findings prove true for multiple sensory systems, suggesting that the ability of sensory input to influence neuron morphology is possibly a universal trait across sensory networks (Knudsen, 2004; Berardi et al., 2015; Dorrego-Rivas and Grubb, 2022). However, the regulation of other features of cell differentiation by external stimuli is much less common. There may be parallels for the melanophore scenario with osteogenic and vasculogenic systems. For example, chondrocyte differentiation of mesenchymal stem cells is triggered by mechanical stress, linking cartilage development to mechanical stimuli (Zuscik et al., 2008). Similarly, maturation and differentiation of endothelial progenitor cells is initiated by shear stress created during blood flow (Kutikhin et al., 2018). In both these cases, it appears populations of undifferentiated precursors are maintained and prepared to respond to mechanical stimulation (Kutikhin et al., 2018; Nishida and Kubota, 2020). A key difference between these systems and our observations of melanophores is that the undifferentiated stem and/or progenitor cells sense and respond to mechanical stimuli directly, whereas in our model a classical sensory neural circuit is involved. We propose that the influence of visual input on perioptic melanophore development reflects a novel mechanism where cell identity is fluid and differentiation is intimately tied to the external environment.

## Data Availability

The datasets presented in this study can be found in online repositories. The names of the repository/repositories and accession number(s) can be found in the article/Supplementary Material.
