# Supplementary material for: Visual input regulates melanophore differentiation
Source: Front Cell Dev Biol. 2024 Aug 20;12:1437613. doi: 10.3389/fcell.2024.1437613 (PMC11368843; doi:10.3389/fcell.2024.1437613)
Supplement: Supplementary file 1 [file DataSheet1.pdf]

Supplementary Material

Visual input regulates melanophore differentiation

Karen Atkinson-Leadbeater[1*], Gabriel E. Bertolesi[2], Sarah McFarlane[2]

*** Correspondence:** Karen Atkinson-Leadbeater: katkinsonleadbeater@mtroyal.ca

1 Supplementary Figures

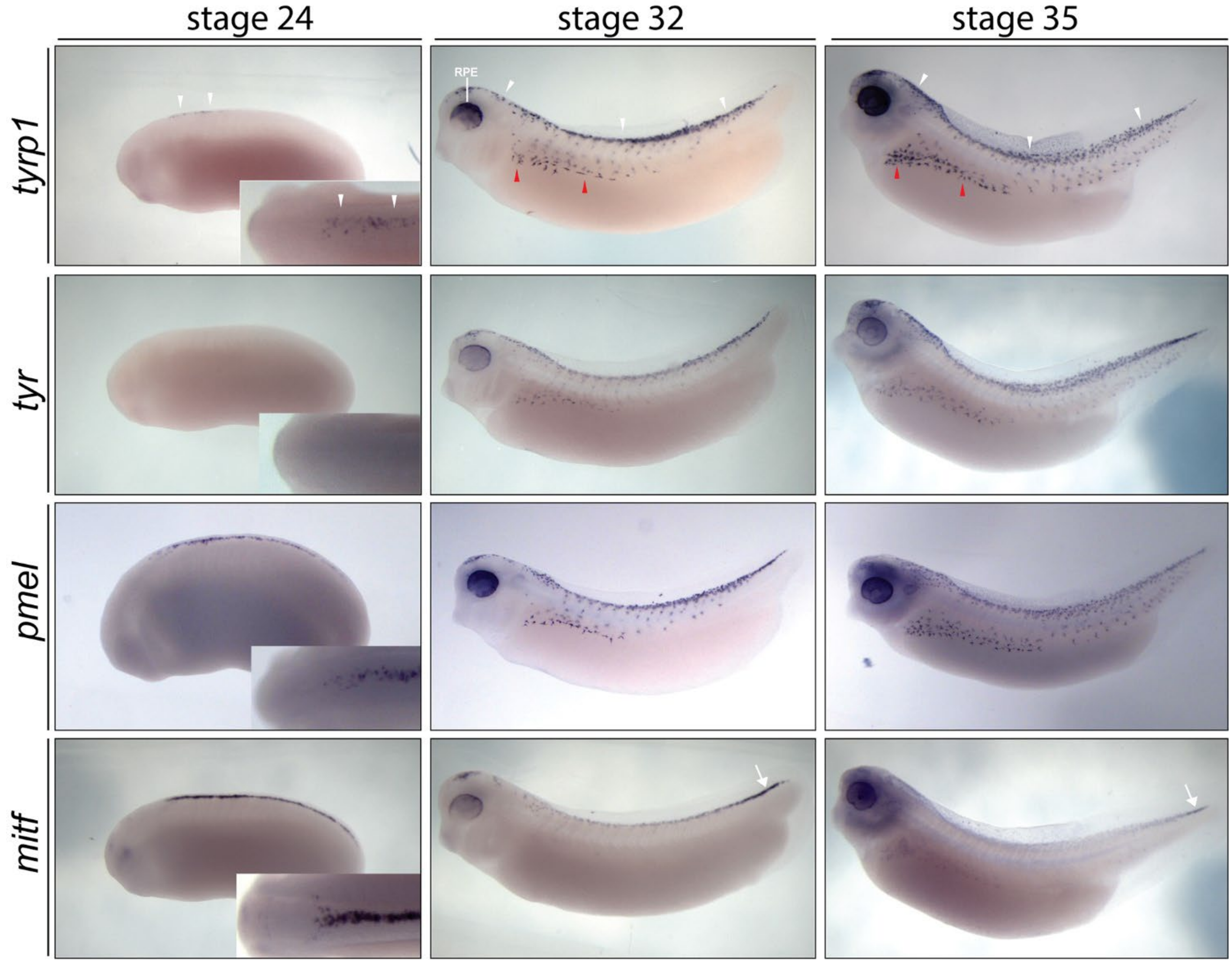

Supplementary Figure 1. Developmental expression patterns of *tyrp1, tyr, pmel*, and *mitf*. WMISH reveals that at stage 24, *tyrp1, pmel,* and *mitf* are expressed along the dorsal midline (white arrowheads, insets provide dorsal view), whereas *tyr* expression is not yet obvious at this stage. By stage 32, all four genes are expressed in the retinal pigmented epithelium (RPE) and along the dorsal

midline from the head to the tail (white arrowheads). Notably, *mitf* expression is diminished along most of the dorsal midline compared to the other genes, with the exception of the tip of the tail (white arrow). In addition, *tyrp1*, *tyr*, and *pmel* show strong expression in the emerging melanophores along the flank (red arrowheads), however, the *mitf* probe does not label these cells. All four genes continue to be expressed in the RPE at stage 35, and *tyrp1*, *tyr*, and *pmel* are expressed in emerging melanophores along the dorsal midline, tail and flank, whereas *mitf* is most strongly expressed in midline cells near the tip of the tail.

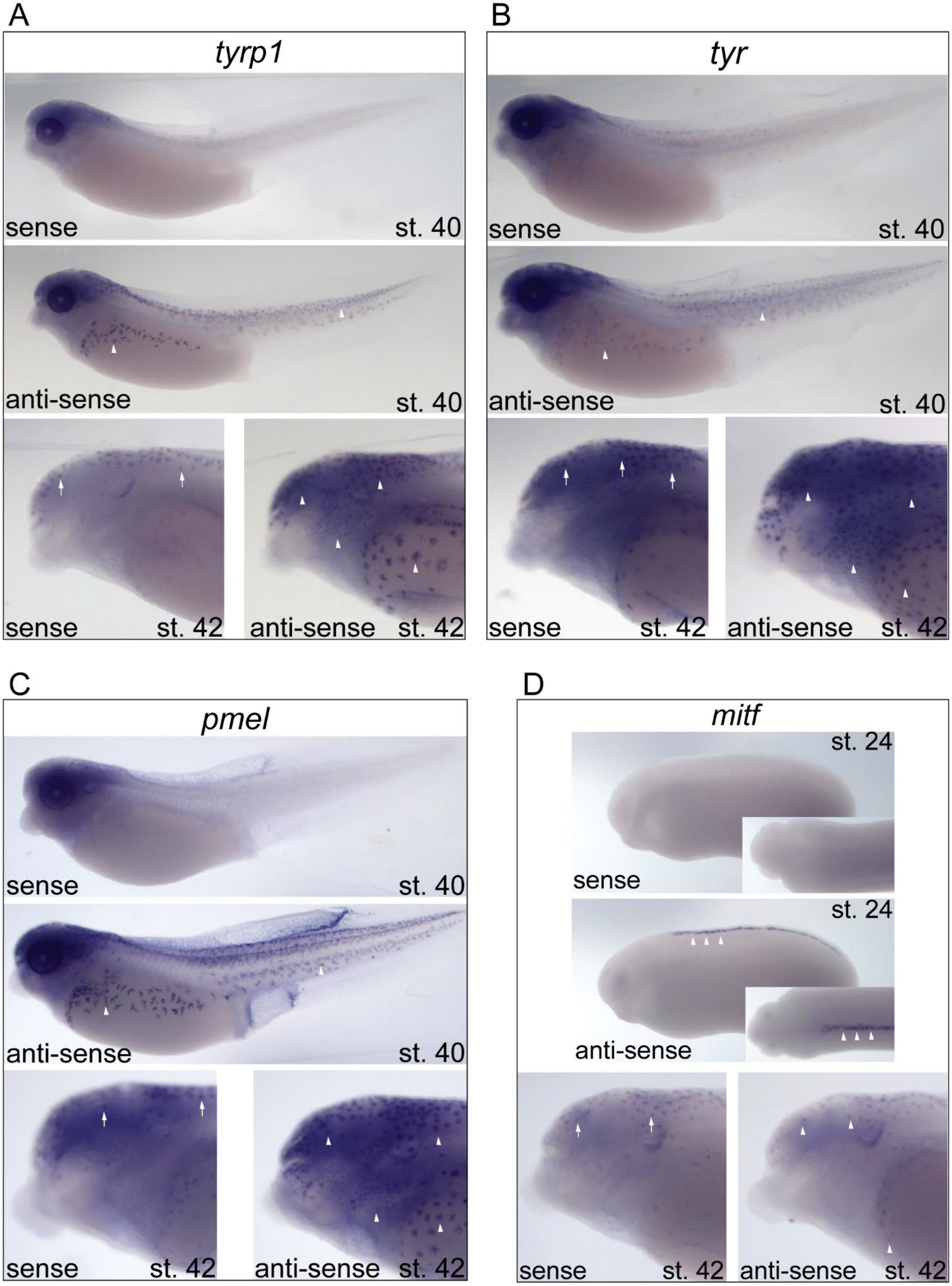
A
tyrp1
sense
st. 40
anti-sense
st. 40
sense
st. 42
anti-sense
st. 42
B
tyr
sense
st. 40
anti-sense
st. 40
sense
st. 42
anti-sense
st. 42
C
pmel
sense
st. 40
anti-sense
st. 40
sense
st. 42
anti-sense
st. 42
D
mitf
st. 24
sense
st. 24
anti-sense
sense
st. 42
anti-sense
st. 42

Supplementary Figure 2. Sense probes do not label melanophores in embryos, but do reveal non-specific staining of some melanophores in larvae. WMISH were performed comparing sense and anti-sense probes at embryonic and larval stages. In stage 40 embryos, antisense probes for *tyrp1*, *tyr*, and *pmel* clearly label flank and tail melanophores (arrowheads), whereas sense probes developed in tandem on sibling embryos do not (tail and flank melanophore label was observed in n=0/9 for sense-*tyrp1* and n=6/6 for antisense-*tyrp1* (**A**), n=0/7 for sense-*tyr* and n=8/8 for antisense-*tyr* (**B**), n=0/8 for sense-*pmel* and n=8/8 for antisense-*pmel* probes (**C**). Similarly, a sense probe for *mitf* failed to label melanoblasts along the dorsal midline in stage 24 and 32 (not shown) embryos, whereas the antisense *mitf* probe clearly labels this population (**D**; dorsal midline staining was observed in n=0/8 for sense-*mitf* and n=8/8 for antisense-*mitf* probes). To test non-specific background staining at later developmental stages, we used larvae fixed 24 h after stage 40 enucleation and PTU treatment. Note: 24 h PTU treatment diminishes pigmentation in new perioptic melanophores arising from enucleation, but leaves existing melanophores darkly pigmented. We observed non-specific background staining in dorsal head and flank melanophores after WMISH with all four sense probes (*arrows*). However, very little non-specific staining was observed in the perioptic region of these larvae: perioptic cell label was observed in n=1/7 larvae for sense-*tyrp1*, n=0/8 for sense-*tyr*, n=4/15 for sense-*pmel*, and n=3/11 for sense-*mitf* probes. Conversely, antisense probes for the melanization genes labeled perioptic cells: n=5/6 for antisense-*tyrp1*, n=7/8, for antisense-*tyr*, n=13/15 for antisense-*pmel* probes. Very few larvae (n=3/15) displayed any perioptic label after WMISH with the antisense-*mitf* probe.
